# Metabolic Pathway Analysis in Chicken Induced by Selenium-Enriched Yeast: Insights from Flavoromics and Metabolomics

**DOI:** 10.3390/foods14234060

**Published:** 2025-11-26

**Authors:** Dan Fei, Min Xie, Daojie Li, Yelan Guang, Yaomin Zhou

**Affiliations:** 1 Institute for Quality, Safety and Standard of Agricultural Products, Jiangxi Academy of Agricultural Sciences, Nanchang 330200, China; feidan1995@163.com (D.F.); ncuskxiemin@163.com (M.X.); lidaojie0723@163.com (D.L.); guangyelan@163.com (Y.G.); 2 Jiangxi Provincial Key Laboratory for Quality and Safety of Agricultural Products, Nanchang 330200, China

**Keywords:** selenium-enriched yeast, chicken, flavors, volatile organic compounds, metabolomics

## Abstract

Flavoromics and metabolomics were used to evaluate the effects of selenium-enriched yeast (SEY) in hen feed on the volatile flavor and nutritional quality indicators in the resulting chicken meats. Volatile organic compounds (VOCs) were analyzed using GC-MS and odor activity value calculations. Ninety-eight VOCs were identified. The treatment group had more abundant VOCs, with mainly increased fat, mushroom, fruit, and vanilla odor. Modulating the nutritional profile of chicken meat through SEY feed can reduce saturated fatty acid levels, increase unsaturated fatty acid levels, and significantly reduce cholesterol levels in Dongxiang green-shell chicken (DX). Metabolomics analysis has elucidated the potential mechanisms, whereby adding SEY to the diet of DX reduces cholesterol levels through secondary bile acid biosynthesis pathways. Its related metabolic changes may also directly or indirectly promote the formation of key substances that give meat its flavor. Adding SEY to the diet of Jingfen laying hens affects the muscle metabolism environment via the propanoate metabolism pathway, increasing the grassy notes of chicken meat while reducing its gamey taste.

## 1. Introduction

Meat flavor is constituted by both taste and olfactory sensations [1]. Taste originates from non-volatile, water-soluble substances, while the aroma is governed by a complex mixture of volatile organic compounds (VOCs) [2]. These VOCs are formed through key biochemical processes in meat, including lipid oxidation, the Maillard reaction, Strecker degradation, and thiamine degradation [3]. The VOCs are often determined by gas chromatography mass spectrometry (GC-MS), which is characterized by qualitative analysis before quantitative analysis [4]. In addition, headspace solid-phase microextraction-gas chromatography mass spectrometry (HS-SPME-GC-MS) is also used for detecting the VOCs [5,6]. The detecting technology integrates collection, extraction, concentration, desorption, and sample injection with the characteristics of small injection volume, convenient operation, high sensitivity, and high reproducibility, while also being solvent-free and non-toxic. Moreover, the odor activity value (OAV) accurately reflects the contribution of the VOCs to the overall aroma. The combination of aroma threshold and OAV theory plays a key role in evaluating the contribution of a VOC to the overall aroma [7]. Metabolomics, a key component of systems biology, has been widely applied in meat and meat products research. It is generally classified into targeted and non-targeted metabolomics, with detection techniques primarily involving mass spectrometry and nuclear magnetic resonance [8]. Non-targeted metabolomics is characterized by high throughput, ultra-sensitivity, wide coverage, and precise characterization. It has been widely applied in chicken research, providing valuable insights into chicken quality and its biochemical processes [9,10,11]. Metabolomics technology can accurately analyze non-volatile compounds in chicken and systematically reveal the dynamic changes in metabolites in chicken processing, storage, and at other stages [12,13]. By using metabolomics to search for, screen, and label the differential metabolites related to chicken quality, differential metabolic molecules that affect chicken quality and establishing corresponding evaluation systems can be explored. The screened differential metabolites can be used as biomarkers for evaluating chicken quality and quality detection.

Chicken meat is the second largest source of consumed meat protein, which is available worldwide and contains lower fat and cholesterol levels compared to red meat [14]. Improving the quality of chicken meat through dietary regulation will help promote chicken consumption and increase the economic benefits of farming. At present, the modulation of chicken nutritional profile and flavor mainly focuses on aspects such as breed, diet, and feeding environment. Breed has an impact on chickens through genetic factors. Compared to fast and large broiler chickens with higher breeding levels, local chickens have better meat flavor, mainly due to the presence of flavor amino acids, nucleotides, reduced sugar, and unsaturated fatty acids in the meat [15]. Different raw material compositions in feed, such as carbohydrates, proteins, trace elements, and so on, can have an impact on meat quality [16]. Farmer et al. found that reducing feeding density is beneficial for enhancing the aroma of chicken meat [17]. Ismail et al. found that feeding garlic powder and phenyl acetic acid in their diet had positive effects on the performance traits and immunological, antioxidant, and physiological status of broilers [18]. Adding some functional ingredients to feed can improve the quality of chicken meat.

Selenium (Se) is an essential trace element that has a large number of biological functions in human and poultry organisms. Adding it to animal feed can enhance muscle succulence and tenderness, while reducing fat and flavor content [19]. Se participates in the formation of glutathione peroxidase (GSH-Px) and is the activity center of enzymes. Glutathione peroxidase can cooperate with superoxide dismutase and catalase to protect cells from damage caused by free radicals, hydrogen peroxide, or lipid peroxide [20]. The enzyme’s activity in the liver or plasma provides valuable insight into the organism’s selenium supply. Previous studies have shown that, in chickens, growth performance, survival, meat quality, and antioxidant protection level are affected by dietary Se status [21]. Many experimental studies have confirmed that selenomethionine- and Se-enriched yeast are the most suitable forms of Se for use in animal nutritional supplements due to their excellent bioavailability and lower toxicity compared to various other forms of Se [22]. Dietary Se supplementation has been shown to increase the egg production rate and antioxidant capacity [23]. Hou et al. showed that selenium-rich brewing yeast improves meat quality in broiler chickens and oxidative stability by activating the glutathione and thioredoxin systems [24].

The effect of selenium-enriched yeast on the quality and flavor of local and commercial chicken meat has not been extensively studied. In this study, Dongxiang green-shell chicken (local chicken breeds in Fuzhou City, Jiangxi Province, China) and Jingfen No. 1 laying hens (commercial chicken) were selected as experimental subjects. The Jingfen No.1 is an excellent egg-laying breed, noted for its high yield, robustness, and pink-shelled eggs. The Dongxiang green-shell chicken is a valued native breed in Jiangxi Province China, distinguished by its unique green-shelled eggs and high-quality meat. Its slower growth results in fine muscle texture, yielding a flavorful, tender, and aromatic product. Through flavoromics and non-targeted metabolomics analysis of VOCs and metabolites in two chicken breeds, the aim of this study is to provide a theoretical basis for selenium-enriched yeast as a feed additive to regulate chicken meat flavor and quality.

## 2. Materials and Methods

### 2.1. Animals and Experimental Treatments

A total of 240 24-week-old laying hens (120 Jingfen No. 1 and 120 Dongxiang green-shell chickens) with similar laying performances and body weights were selected from commercial farms in Jiangxi Province. Hens of each breed were randomly allocated into control and treatment groups (*n* = 60 per group), further divided into six subgroups (ten hens/subgroup) with equal representation of cage positions. Birds were maintained in an environmentally controlled facility (23–25 °C, 60% RH) with a 16 h:8 h light/dark schedule. and ad libitum access to feed and water. The basal diet, except for Se, (Table S1) was formulated according to the National Research Council requirements as described by Zhao et al. [25]. Control groups (JF and DX) received the basal diet, while treatment groups (JF-Se and DX-Se) were supplemented with 0.4 mg/kg of selenium-enriched yeast. After 60 days of feeding, six randomly selected hens per group were fasted for 12 h and humanely euthanized by cervical dislocation following AVMA guidelines.

### 2.2. Sample Collection and Preparation

The chicken breast of each hen was collected, then shipped in ice boxes to the lab. Upon arrival, each chicken breast sample was homogenized and repackaged, then refrigerated at −20 °C.

### 2.3. Chemicals and Reagents

Petroleum ether, formic acid, ammonium acetate, methanol, ethanol, ether, potassium hydroxide, sodium sulfate, petroleum ether, hydrochloric acid, n-Alkanes (C_9_-C_30_), 2-octanol (purity ≥ 99.5%), and cholesterol (purity ≥ 99%) were purchased from Sigma-Aldrich (Shanghai, China). The experimental water was ultrapure water, which was made using the Milli-Q purification system (Millipore, Molsheim, France). Selenium-enriched yeast purchased from Jiangxi Xinweian Agricultural Development Co., Ltd. (Nanchang, China).

### 2.4. Extraction of VOCs Using Headspace Solid-Phase Microextraction (HS-SPME)

To ensure the effective extraction of VOCs, the key parameters of HS-SPME were optimized. The parameters optimized included the incubation temperature and time, the selection of the SPME fiber coating, the extraction time, and the thermal desorption conditions. These parameters were determined through a series of single-factor experiments to achieve optimal extraction efficiency. Based on the optimization results, the following procedure was employed for sample analysis. A precise amount (1.0 g) of chicken breast homogenate was weighed and placed into a 20 mL headspace (HS) vial. The vial was incubated in a water bath at 50 °C for 30 min. VOCs were then extracted using a SPME fiber (50/120 μm DVB/CAR/PDMS; CTC Analytics, Zwingen, Switzerland) exposed to the vial headspace at 50 °C for 10 min. Following extraction, the fiber was inserted into the GC-MS injector port for thermal desorption at 270 °C for 5 min to release the adsorbed analytes for chromatographic analysis.

### 2.5. GC-MS Analysis

VOCs were analyzed using a gas chromatography triple quadrupole tandem mass spectrometer (GC-MS-TQ8040, Shimadzu, Japan) equipped with a DB-WAX capillary column (30 m × 0.25 mm × 0.25 μm, Shimadzu, Kyoto, Japan). The injector temperature was set at 250 °C and the carrier gas (helium, 99.999%) flow rate was maintained at 1.0 mL/min. The oven temperature program was as follows: initial hold at 50 °C for 5 min, ramped to 250 °C at 10 °C/min, and held for 10 min (total run time: 35 min). Electron ionization (EI) was performed at 70 eV with an ion source temperature of 200 °C. Mass spectra were acquired in scan mode over a range of 35–400 m/z. Instrumental parameters followed the method described by Fei et al. [26].

### 2.6. Analysis of VOCs

Qualitative analysis was performed as follows. VOCs were identified by matching their mass spectra and retention index (RI) against reference databases, including the NIST 17, AROMA spectral database, and Shimadzu Off-flavor database. Retention indices were calculated using n-alkanes (C_9_-C_30_) analyzed under identical GC-MS conditions [27].

Relative quantitative analysis was performed using the internal standard method for quantification, setting the factor of the internal standard as one, and using 2-octanol as an internal standard. The unknown compound content is calculated using the following equation:

$$C_{i}=\frac{C_{0}*V_{0}*A_{i}}{A_{0}*m}$$

where *C_i_* is the content of the unknown component (ng/g); *C*_0_ is the mass concentration of the internal standard; *V*_0_ is the injection volume of the internal standard (μL); *A_i_* is the peak area of the unknown component; *A*_0_ is the peak area of the internal standard; and m is the sample mass.

### 2.7. Calculation of Odor Active Value (OAV)

The OAV is calculated using the following equation [28]:

$$OAV=\frac{C_{i}}{OT_{i}}$$

where *C_i_* is the concentration of the VOCs (μg/g); *OT_i_* is the odor threshold of the VOCs in water, which was obtained from book [29].

### 2.8. Amino Acid Analysis

Accurately weigh 0.2 g of chicken meat into a stoppered test tube, add 10 mL of 50% hydrochloric acid solution, and hydrolyze at 110 °C for 22 h. Transfer the hydrolysate to a 100 mL volumetric flask, dilute to volume with ultrapure water, and mix thoroughly. Pipette 5 mL of the diluted solution, filter it, then transfer 1 mL of the filtrate to a small beaker. Evaporate the filtrate to dryness under reduced pressure at 60 °C, and redissolve the residue in 1 mL of 0.02 M hydrochloric acid solution. After membrane filtration, analyze the solution using an amino acid analyzer.

### 2.9. Fatty Acids Analysis

Fatty acid composition was determined following the method of Shakoor et al. (2025) with modifications [30]. Briefly, 2 g of chicken meat was homogenized with 10 mL petroleum ether and extracted by ultrasonication (25 °C, 30 min). The organic phase was collected and esterified with 5 mL of methanolic sodium hydroxide (0.5 M) under ultrasonication (25 °C, 10 min). The mixture was then dehydrated with 1 g of anhydrous sodium sulfate (5 min standing), and the supernatant was collected. Fatty acid methyl esters (FAMEs) were filtered through a 0.22 μm nylon membrane prior to GC-MS analysis. GC-MS 7890B (Agilent Technologies, Santa Clara, CA, USA) was used with a DM-2560 column (100 m × 250 μm × 0.2 μm, DiKMA, Beijing, China). The oven temperature was set at 100 °C for 4 min and then raised to 240 °C at 3 °C/min. The injection volume was 2 μL, using 1.2 mL/min carrier gas (hydrogen). FID detection was set at 250 °C. FAMEs were quantified as a percentage of fatty acid peak area.

### 2.10. Cholesterol Analysis

Cholesterol content was determined using an Agilent 1260 HPLC system with external standard calibration. Briefly, 500 mg of chicken meat was saponified with anhydrous ethanol–potassium hydroxide solution (1:1, *v*/*v*), followed by lipid extraction using petroleum ether–anhydrous ether (3:1, *v*/*v*). The organic phase was concentrated to dryness under nitrogen, reconstituted in anhydrous ethanol, and filtered (0.22 μm) prior to analysis. Chromatographic separation was achieved on a C18 column (4.6 × 150 mm, 5 μm; Agilent, USA) maintained at 38 °C. The isocratic mobile phase (methanol) was delivered at 1.0 mL/min with detection at 205 nm. A 10 μL aliquot was injected for each analysis. Quantification was performed using external cholesterol standards with linear calibration.

### 2.11. Untargeted Metabolomics Analysis

Metabolites’ extraction and ultra-high performance liquid chromatography-tandem mass spectrometry (UHPLC-MS/MS) analysis. For the metabolite analysis, chicken meat samples (100 mg) were flash frozen in liquid nitrogen and homogenized before being extracted with 1 mL of ice-cold 80% methanol containing 0.1% formic acid through vigorous vortexing (1 min) followed by ice incubation (5 min). After centrifugation (14,000× *g*, 4 °C, 5 min), the supernatant was diluted with ultrapure water to 53% methanol concentration and centrifuged again (14,000× *g*, 4 °C, 10 min). LC-MS/MS analysis was performed using a Thermo Scientific Vanquish UHPLC system coupled to an Orbitrap Q Exactive™ HF mass spectrometer equipped with a Hypesil Gold C18 column (100 × 2.1 mm, 1.9 μm, Thermo Fisher Scientific, Waltham, MA, USA). Separation employed a 17 min gradient (0.2 mL/min) with mobile phases consisting of (A) 0.1% formic acid in water (positive mode) or 5 mM ammonium acetate (pH 9.0, negative mode) and (B) methanol, using the following profile: 2% B (0–1.5 min), linear increase to 100% B (1.5–13.5 min), hold (13.5–15.5 min), return to initial conditions (15.6 min), and re-equilibration (15.6–17 min). MS detection utilized both polarity modes with spray voltage (3.2 kV), capillary temperature (320 °C), sheath gas (40 arb), and auxiliary gas (10 arb). A quality control (QC) sample was prepared by pooling equal volumes of all experimental samples. This QC sample was utilized to monitor instrument performance, equilibrate the UHPLC-MS/MS system, assess the overall system stability throughout the experimental process, and conduct data quality control analysis.

Data processing. The raw data files generated by UHPLC-MS/MS were processed using the Compound Discoverer 3.1 (CD3.1, Thermo Fisher) to perform peak alignment, peak picking, and quantitation for each metabolite. The main parameters were set as follows: retention time tolerance, 0.2 min; actual mass tolerance, 5 ppm; signal intensity tolerance, 30%; signal/noise ratio, 3; and minimum intensity, 100,000. After that, peak intensities were normalized to the total spectral intensity. The normalized data was used to predict the molecular formula based on additive ions, molecular ion peaks, and fragment ions. Then, peaks were matched with the mzCloud, mzVault, and MassList database to obtain accurate and relative quantitative results. Data were normally distributed, and normal transformations were attempted using the area normalization method. The raw data from all samples were processed by performing peak alignment across all samples and normalizing the peak areas using the first sample as a reference to ensure more accurate quantification. Metabolites were then identified by comparing the accurate mass (within a 10 ppm tolerance) and adduct ion information against a high-quality MS/MS spectral database.

Bioinformation analysis. The Orthogonal projection to latent structures discriminant analysis (OPLS-DA) model was applied in comparison groups using R package models (http://www.r-project.org/). Then, the OPLS-DA model was further validated by cross-validation and permutation. A variable importance in the projection (VIP) score of the OPLS-DA model was applied to rank the metabolites that best distinguished between the two groups. A T-test was also used as a univariate analysis for screening differential metabolites. Those with a *p*-value < 0.05 and VIP > 1 were considered differential metabolites between two groups. Metabolites were mapped to Kyoto Encyclopedia of Genes and Genomes (KEGG) metabolic pathways for annotation and enrichment analysis.

### 2.12. Data Analysis

All data are expressed as mean ± standard deviation. One-way analysis of variance analysis (*n* = 6) [31] and Tukey’s test (*p* < 0.05) were used to determine the significant differences in VOCs, fatty acids, and amino acids across groups. Multivariate analyses including principal component analysis (PCA) [32], heatmap visualization, and partial least squares discriminant analysis (PLS-DA) were conducted using MetaboAnalyst 6.0. Variable importance in projection (VIP) values were obtained based on the PLS-DA model. The compounds with VIP > 1.0 and *p* < 0.05 were selected as differential metabolites. Metabolic pathway analysis was performed using the Kyoto Encyclopedia of Genes and Genomes (KEGG) database. Spearman’s correlation coefficients between differential metabolites and key nutritional components (characteristic VOCs, essential nutrients, and amino acids) were calculated and visualized using Origin (OriginLab Corp., Northampton, MA, USA).

## 3. Results and Discussion

### 3.1. VOCs Analysis

The total ion chromatograms of VOCs were obtained by HS-SPME-GC-MS. Combined with the NIST database, Shimadzu off favor database, and AROMA spectral database, 98 VOCs were identified in chickens from four groups (Table 1 and Table S2), including 24 alcohols, 18 aldehydes, 24 esters, 12 ketones, 6 acids, 7 aromatic hydrocarbons, 4 heterocycles, 1 phenols and 2 cycloolefins. The concentration of each VOC was determined by the internal standard method, and the data is presented as mean ± standard deviation. The standard deviation values are noticeable. This is primarily attributed to the inherent complexity of the chicken meat matrix and the trace-level, labile nature of flavor compounds. Biological variability between individuals, minor heterogeneity within the homogenized sample, and the competitive adsorption process of HS-SPME in such a complex matrix are well-documented sources of variance in food flavor analysis. To mitigate this, we used a pooled and thoroughly homogenized sample source and strictly controlled all analytical parameters. Despite the absolute variance, the relative profiles and statistically significant differences between treatment groups were clear and reproducible. Sixty-six VOCs were identified in the DX-Se group, fifty-eight VOCs were identified in the DX group, sixty-seven VOCs were identified in the JF-Se group, and sixty-one the VOCs were identified in JF group.

**Figure 1:**
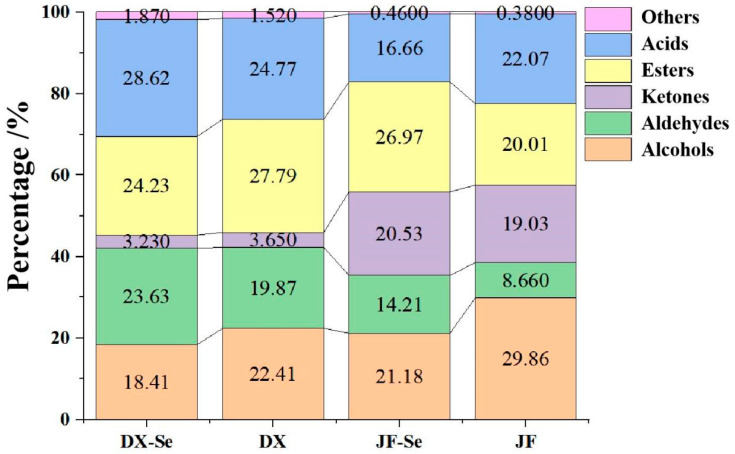
Proportion of VOCs in chicken. DX-Se: treatment group of Dongxiang green-shell chicken. DX: control group of Dongxiang green-shell chicken. JF-Se: treatment group of Jingfen laying hens. JF: control group of Jingfen laying hens.

The profile of flavor substances in chicken is dominated by alcohols, aldehydes, and esters, a result that aligns with the existing literature [33]. Alcohols have a lower sensory threshold, which helps chicken form characteristic flavors. In our study, the content of 1-pentanol (17), 1-hexanol (25), and 1-octen-3-ol (37) were abundant. 1-octene-3-ol is the most important alcohol in chicken, which was formed by the oxidation of linoleic acid, and has a low sensory threshold and a strong mushroom odor. Research has found that the expression levels of *HSP90AA1*, a member of the heat shock protein (HSP) family, and non-receptor protein tyrosine phosphatase 9 (*PTPN9*) genes were significantly positively correlated with the content of 1-octen-3-ol [34]. According to Merlo et al., hexanol originates from the reduction reaction of hexanal, while pentanol originates from the degradation of lipid hydroperoxides [35]. Pentanol can provide a stimulating and strong vinegar odor. Ketones could be produced through thermal degradation, polyunsaturated fatty acids oxidization, amino acid degradation, microorganism oxidization, and Maillard reactions [36]. As shown in Figure 1, aldehydes were rich VOCs in chicken, and have a lower sensory threshold, thus playing an important role in the overall flavor of chicken. The content of hexanal (2), octanal (22), nonanal (31), and neral (53) were higher in the DX-Se group than those in DX group. Motram et al. found that aldehydes with five to nine carbon atoms have a fragrant, oily, and greasy odor [37]. In this study, selenium-enriched yeast was added to the hens’ diets and the relative content of these aldehydes were increased.

Ketones mainly originated from fat oxidation, especially 2-ketones, which are considered to have a significant contribution to meat flavor [38]. According to reports, 2-pentanone, 2-heptanone, and 2-decanone exhibit an odor similar to ether, butter, and cheese [38]. In this study, 2-nonanone (29) was abundant in the JF-Se group, which was described as with a hot milk, soap, and green odor. 2-undecanone (46) was abundant in JF-Se and JF groups, which was described as the orange, fresh, green odor. Previous published research suggested that the types and contents of volatile compounds could be regulated by organic Se, especially aldehydes and ketones, which affected the changes in muscle flavor [39]. The high production of aldehydes and ketones may be due to the influence of organic selenium on early-stage metabolism and enhanced fat decomposition in the body [40]. Some heterocyclic compounds were also detected in four groups of chickens. Approximately 90% of the aromatic substances are derived from lipid oxidation, followed by the Maillard reaction and thiamine degradation. Although the latter two reactions produce less than 10% of these volatile substances, their influence on meat flavor cannot be underestimated, as they mainly produce carboxyl compounds, sulfur compounds, nitrogen, and oxygen heterocyclic compounds. In the present study, 2-methylpyridine (15), 4-methylthiazole (20), 4,5-dimethylthiazole (26), and benzothiazole (82) were detected.

Overall, in DX-Se and DX groups, adding selenium-enriched yeast to the diet made more VOCs in chicken. Among them, the content of aldehydes, acids, and heterocycles increased, while the content of esters, ketones, and alcohols decreased. Similarly, in JF-Se and JF groups, more abundant VOCs are derived from adding selenium-enriched yeast to the diet. The content of esters, aldehydes, ketones, and heterocycles increased, while the content of acids and alcohols decreased. There were differences in the effects of selenium-enriched yeast on VOCs of different chicken breeds.

### 3.2. Chemometric Analysis of VOCs

PCA was applied to analyze the differences in VOCs among different chicken meat samples. In the scores plot (Figure S1A), each point represents an individual sample, with points of the same color belonging to the same group. The distance between points reflects the degree of dissimilarity between samples—greater distances indicate lower similarity in VOCs profiles. The first two principal components accounted for 52% and 26.9% of the total variance, respectively. In the score plot, the DX-Se group was clearly separated from the DX group, suggesting that dietary supplementation with selenium-enriched yeast significantly altered the VOC characteristics of chicken meat. Moreover, the DX-Se, DX, JF-Se, and JF groups were all distinctly separated from one another, indicating notable differences in VOCs among chickens from different laying hen breeds. The chicken loadings plot (Figure S1B) showed that 2-heptanone (10), heptyl acetate (27), trans-p-methane-8-thiol-3-one (70), and caryophyllene oxide (85) had higher positive loads on PC1, which corresponded to the JF group in the PCA score plot, indicating that these VOCs were more abundant in the JF group. Similarly, trans-2-heptenal (24), nonyl acetate (44), naphthalene (61), 12-methyltridecanal (75), butylated hydroxytoluene (78), tetradecanal (79), maltol (81), (E)-2-hexenoic acid (83), and 1-tetradecanol (95) had a higher negative load on PC2, corresponding to DX-Se group. In the same way, 2-Methylbutyl acetate (3), hexyl acetate (19), 4-methylthiazole (20), neodihydrocarveol (58), neryl acetate (59), geranyl isobutyrate (67), and alpha-ionone (71) were more abundant in the DX group, and 2-nonanone (29), (Z)-linalool oxide (35), (E)-2-nonenal (42), terpinen-4-ol (45), (E,E)-2,4-nonadienal (55), and 3-methyl-2,4-nonanedione (57) were more abundant in JF-Se group.

**Figure 2:**
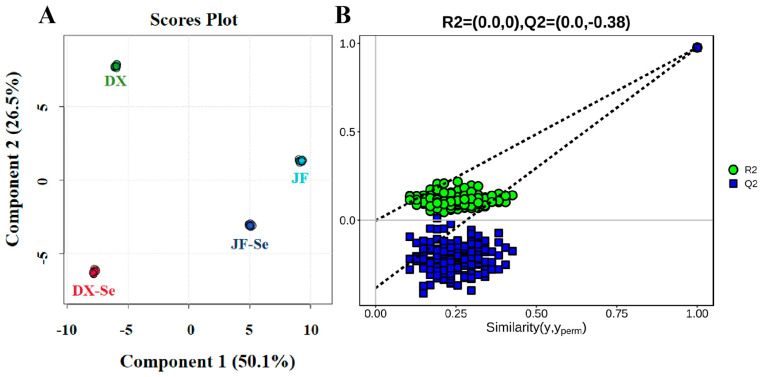
Partial least squares discriminant analysis (PLS-DA) score plot of VOCs in chicken coming from different groups (**A**); permutation test of the PLS-DA model (**B**). DX-Se: treatment group of Dongxiang green-shell chicken. DX: control group of Dongxiang green-shell chicken. JF-Se: treatment group of Jingfen laying hens. JF: control group of Jingfen laying hens.

Compared to unsupervised PCA, PLS-DA was effective in distinguishing observations between groups and was able to find variables that affected differences between groups. As shown in Figure 2A, R^2^ and Q^2^ were 0.977 and 0.976, respectively, which indicated a high accuracy and model stability, as well as its reliability in explaining sample variations [41]. Subsequently, the accuracy of the model was tested with a 200 permutation test (Figure 2B), and there was no overfitting for different chickens.

### 3.3. Characteristic VOCs Analysis

**Figure 3:**
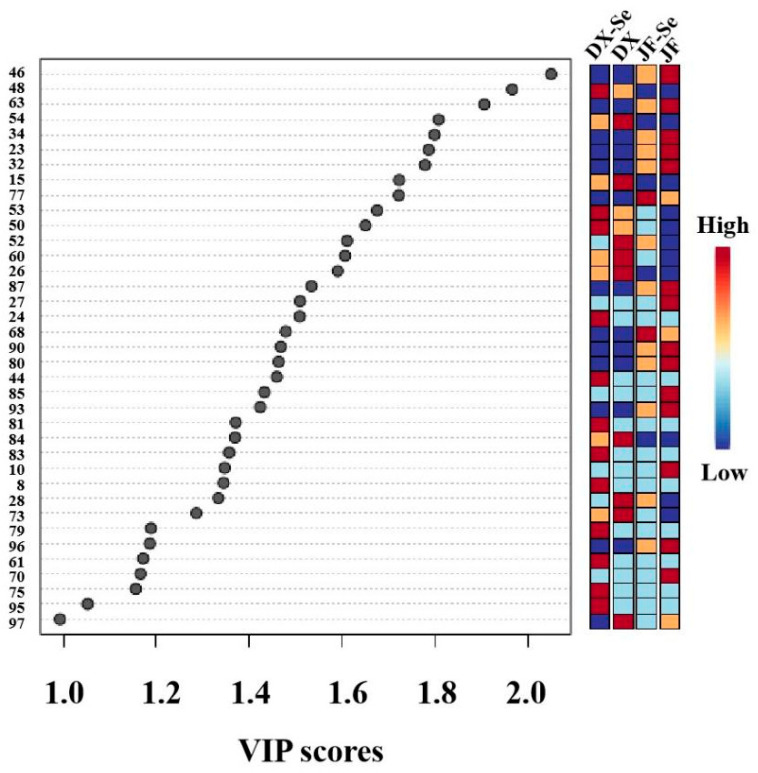
Variable importance on projection (VIP) scores from the PLS-DA model. DX-Se: treatment group of Dongxiang green-shell chicken. DX: control group of Dongxiang green-shell chicken. JF-Se: treatment group of Jingfen laying hens. JF: control group of Jingfen laying hens.

The value of variable importance in projection (VIP) reflects the contribution of each variable to the classification of the samples [42]. The VIP > 1 and *p* < 0.05 compounds were selected as differential markers. Based on the result in Figure 3 and combined with significant difference analysis, a total of 32 significant difference compounds were identified in the chicken, including six alcohols (trans-2-penten-1-ol, cis-3-hexen-1-ol, 1-undecanol, 1-dodecanol, (E)-2-dodecen-1-ol, and 1-tetradecanol), eight aldehydes (trans-2-heptenal, trans-2-octenal, (Z)-2-decenal, neral, geranial, 12-methyltridecanal, tetradecanal, and pentadecanal), six ketones (2-heptanone, trans-3-octen-2-one, 2-undecanone, cis-geranylacetone, trans-p-methane-8-thiol-3-one, and beta-Ionone), seven esters (heptyl acetate, nonyl acetate, (Z)-3-hexenyl hexanoate, decyl acetate, linalyl butyrate, gamma-octalactone, and caryophyllene oxide), one acid ((E)-2-hexenoic acid), and four other compounds (alpha-terpinene, 4,5-dimethylthiazole, naphthalene, and maltol). These substances can be used as marker compounds to distinguish the four groups of chickens studied in this paper.

VOCs are important indicators to evaluate the quality of meat, which are generated by the complex biochemical changes in the inherent components in meat, including lipid oxidation, Strecker degradation, Maillard reactions, and thiamine degradation [43]. In this study, the OAV method was used to evaluate the VOCs identified in the chickens of four groups. A total of 21 VOCs in chicken were available for OAV calculation (Table 2); 1-pentanol (balsamic), 1-hexanol (resin, flower, green), nonanal (fat, citrus, green), 1-heptanol (chemical, green), and 1-octanol (chemical, metal, burnt) maintained high OAVs in chickens from four groups. In the DX-Se and DX groups, the OAVs of 1-pentanol, 1-hexanol, 1-heptanol, and 1-octanol were above 1000. Compounds with 100 < OAV < 1000 included hexanal, nonanal, 1-nonanol, and geranial. Nonanal and hexanal are mainly produced from the oxidization of linoleic acid, which provides chicken flavors similar to plants and fats. Similarly, in the JF-Se group, the OAVs of 1-hexanol, nonanal, 1-heptanol, 1-octanol, and 1-octen-3-ol were above 1000. Compounds with 100 < OAV < 1000 included 1-pentanol, 2-nonanone, 1-nonanol, and geranial. In the JF-Se group, the OAVs of 1-pentanol, octanal, 1-hexanol, nonanal, 1-heptanol, 1-octanol, and 1-octen-3-ol were above 1000. Compounds with a 100 < OAV < 1000 included hexanal and 1-nonanol.

Alcohols are mainly produced by the enzymatic degradation of linoleic acid by lipids in meat under the action of lipoxygenase and hydroperoxidase, and then are oxidized under the action of linoleic acid-degrading enzymes [44]. Alcohols have a lower sensory threshold, which helps chickens form characteristic flavors. 1-Octene-3-ol is one of the most important alcohols in chicken, formed by the oxidation of linoleic acid, with a lower sensory threshold and a strong mushroom-like odor. 1-Hexanol originates from the reduction reaction of hexanal, while 1-pentanol also originates from the degradation of lipid hydroperoxides, among which pentanol can provide a stimulating and strong vinegar odor. 1-Heptanol and 1-octanol are mainly responsible for chemical, green, metal, and burnt odors. Aldehydes are generally produced by fat oxidation with a lower sensory threshold than alcohols and play an irreplaceable role in the composition of meat flavor in chicken [45]. Nonanal is mainly responsible for fat, citrus, and green odors. Overall, in this study, VOCs in the selenium-enriched yeast groups were more abundant than those in the control groups.

### 3.4. Fatty Acid Profiles and Cholesterol Analysis

Table 3 provides insights into the effects of selenium-enriched yeast as a feed additive on the fatty acid compositions of chicken. All samples included 27 different fatty acids that were categorized as saturated fatty acids (SFAs), monounsaturated fatty acids (MUFAs), and polyunsaturated fatty acids (PUFAs). In the DX-Se group and DX group, the content of SFA was 37.69% and 41.04%, respectively, with predominantly palmitic acid (C16:0) and stearic acid (C18:0). Compared with the DX group, the MUFA content and PUFA content in the DX-Se group increased by 2.28% and 1.08%. In MUFA, oleic acid and palmitoleic acid are the main components, while in PUFA, linoleic acid is the main component. Similarly, in the JF-Se group and JF group, the content of SFA was 40.67% and 45.22%, respectively, with predominantly palmitic acid (C16:0) and stearic acid (C18:0). Compared with the JF group, the MUFA content and PUFA content in the JF-Se group increased by 3.80% and 0.74%. In this study, both treatment groups showed a decrease in SFA content and an increase in MUFA and PUFA content compared to the control group. This indicated that modulating fatty acid composition in chicken meat through selenium-enriched yeast feed can reduce the content of SFA and increase the content of UFA in chicken. SFA can increase cholesterol, mainly palmitic acid (C16:0) and stearic acid (C18:0), which are supposed to be the cause of cancer and coronary heart disease. In contrast, unsaturated fatty acids (UFA) can improve the flavor and appeal of meat products and offer health benefits [46]. PUFAs are important precursors of volatile substances [47].

**Figure 4:**
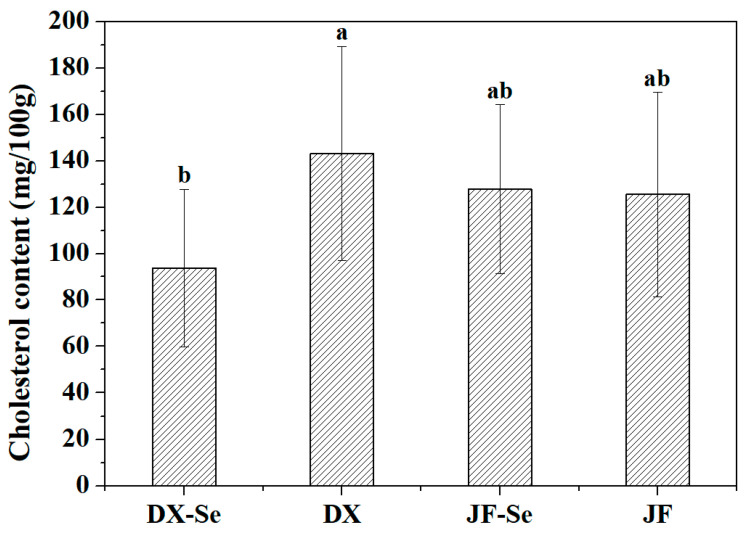
The content of cholesterol (*n* = 6). DX-Se: treatment group of Dongxiang green-shell chicken. DX: control group of Dongxiang green-shell chicken. JF-Se: treatment group of Jingfen laying hens. JF: control group of Jingfen laying hens. Different letters in the row indicate a significant different (*p* < 0.05).

Cholesterol is a steroid lipid that plays an important role in animal metabolism. It is a precursor to bile acids, hormones, and vitamin D, and is widely present in animal derived foods such as meat, eggs, seafood, and dairy products [48]. As shown in Figure 4, the cholesterol content in the DX-Se group was significantly reduced (*p* < 0.05). There was not much difference in cholesterol content between the JF-Se group and the control group. Liver is an important organ for lipid metabolism in the body, and the conversion of cholesterol into bile acids in the liver is an important pathway for cholesterol metabolism. Cholesterol metabolism in the body is regulated by a series of enzymes, among which CYP7A1 is the rate-limiting enzyme for bile acid synthesis and plays an important role in maintaining cholesterol stability in the body. The SREBP-1c gene belongs to the steroid regulatory element binding protein family, which can promote fatty acid production and synthesize cholesterol esters with cholesterol, thereby consuming cholesterol [49]. HMGCR is the rate-limiting enzyme in the endogenous cholesterol synthesis process [50,51]. Studies have shown that adding selenium sources to feed has no significant effect on the blood cholesterol and triglyceride content of laying hens [52]. In this study, selenium-enriched yeast significantly reduced cholesterol in local chicken breeds but had no significant effect on commercial chickens. It can be seen that the regulation of chicken quality by selenium-enriched yeast is influenced by the variety.

### 3.5. Amino Acids Analysis

The amino acids measured included bitter amino acids (leucine, phenylalanine, arginine, lsoleucine, tyrosine, valine, histidine, and lysine), umami amino acids (aspartic acid and glutamic acid), sweet amino acids (serine, alanine, threonine, glycine, and proline), and sulfur-containing amino acids (metthionine) [30]. The contents of amino acids in chickens from different groups are shown in Table 4. The results showed that there was no significant difference in the content of the 16 amino acids between the DX-Se group and DX group. In the JF-Se group, the content of phenylalanine (Phe) and serine (Ser) was significantly increased (*p* < 0.05) compared to those in the JF group. Amino acids play critical roles in human physiological functions, serving as fundamental components for protein synthesis and metabolic regulation. The amino acid profile, particularly the composition of essential amino acids (EAAs), represents a key metric for assessing meat nutritional quality. EAAs such as lysine and leucine must be obtained through dietary sources as they cannot be endogenously synthesized, whereas non-essential amino acids (NEAAs) can be produced metabolically. The nutritional value of dietary protein is largely determined by the absolute content, proportion, and balance of EAAs. As shown in Table 4, all experimental groups exhibited EAA/TAA (total amino acids) and EAA/NEAA ratios exceeding the FAO/WHO ideal protein standards (40% and 60%, respectively; WHO, 1973) [53]. Compared to the control group, the treatment group showed an increase in EAA/TAA and EAA/NEAA values. These findings suggest that dietary selenium supplementation effectively optimizes the amino acid profile in chicken meat, potentially increasing its nutritional value for human consumption.

### 3.6. Metabolomics Analysis

**Figure 5:**
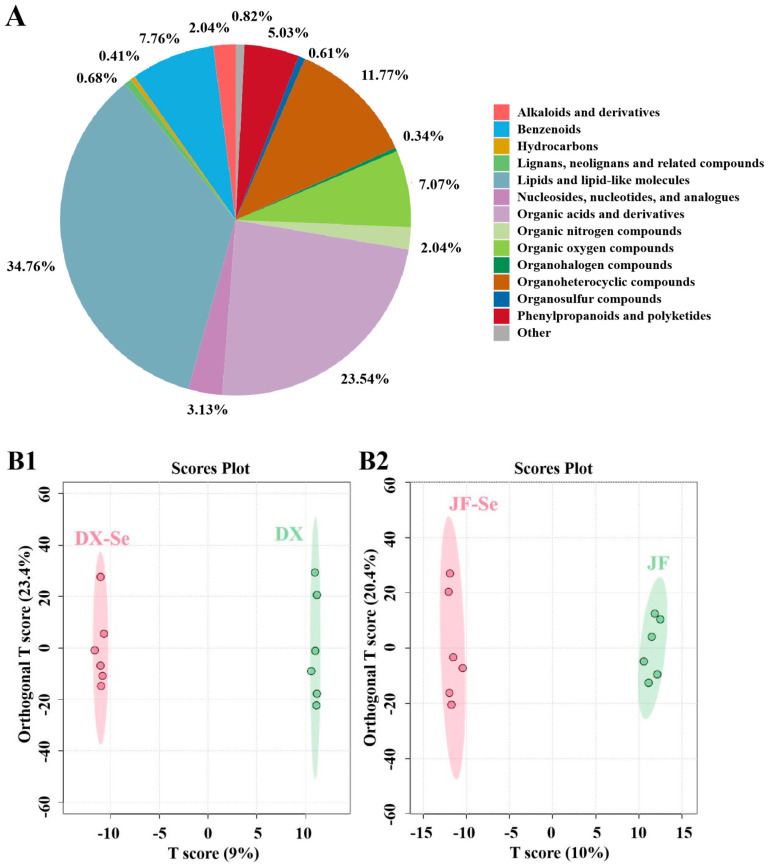

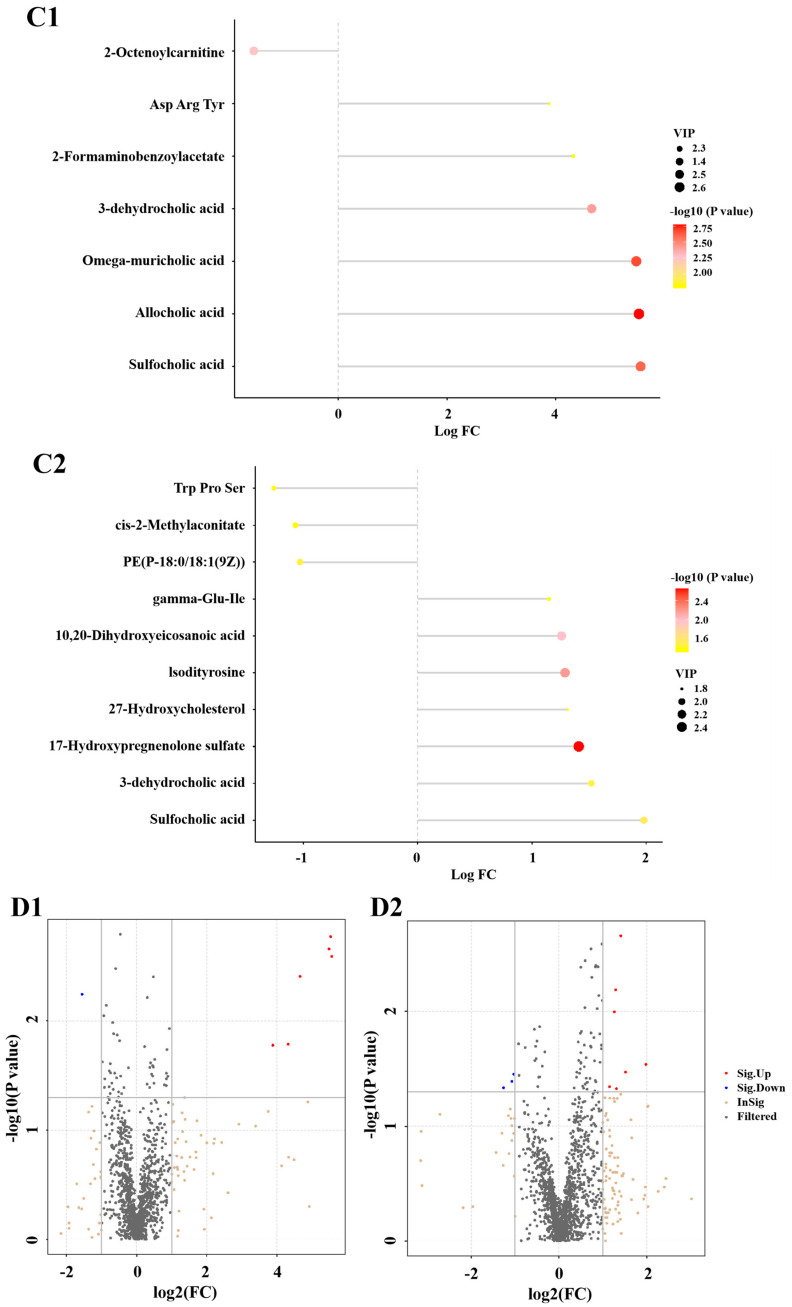
Metabolomics analysis (*n* = 6): pie chart of metabolite classification (**A**); score plot of OPLS-DA (**B1**,**B2**); differential metabolites of chicken meat from Dongxiang green-shell chicken (**C1**); differential metabolites of chicken meat from Jingfen No. 1 laying hen (**C2**); volcanic diagram of differential metabolites of chicken meat from Dongxiang green-shell chicken (**D1**); and volcanic diagram of differential metabolites of chicken meat from Jingfen No. 1 laying hen (**D2**). DX-Se: treatment group of Dongxiang green-shell chicken. DX: control group of Dongxiang green-shell chicken. JF-Se: treatment group of Jingfen laying hens. JF: control group of Jingfen laying hens.

To comprehend the impact of selenium-enriched yeast as a feed additive on water-soluble metabolites, metabolomics analyses were conducted. A total of 1470 water-soluble metabolites were identified in the chickens, 788 and 682 in the positive and negative ion modes, respectively, with lipids and lipid-like molecules being the most predominant, followed by organic acids and derivatives, organoheterocyclic compounds, benzenoids, and organic oxygen compounds (Figure 5A). The OPLS-DA, a regression-based supervised pattern recognition technique, was then applied to the metabolomic dataset (Figure 5(B1,B2)). This method enhances the identification of characteristic variables by differentiating between the predictive and orthogonal components. No crossover points were observed between the control groups and the treatment group in two hen breeds for non-volatile components. The non-volatile components in chicken meat fed with selenium-rich feed were clearly separated. To better understand the effect of selenium-enriched yeast as a feed additive on the non-volatile components in chicken meat, OPLS-DA analysis was performed on the samples, identifying differential metabolites based on fold change (FC) and VIP values. As shown in Figure 5(C1,C2,D1,D2), seven differential metabolites (six up-regulated and one down-regulated) between the DX-Se and DX groups were found using OPLS-DA screening with VIP > 1 and *p* < 0.05 as the criteria. Similarly, ten differential metabolites (seven up-regulated and three down-regulated) between the JF-Se and JF groups were found. Most of the non-volatile metabolites were up-regulated in all treatment groups compared to control groups, suggesting that the production and transformation of non-volatile metabolites were dominant during the treating.

**Figure 6:**
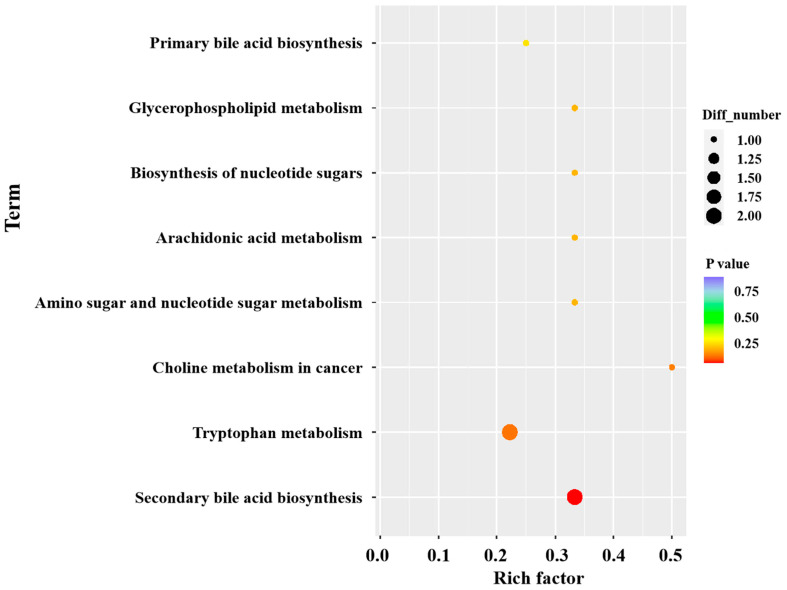
KEGG pathway analysis of chicken meat from Dongxiang green-shell chicken.

KEGG pathway analysis of the differential metabolites revealed nine metabolic pathways enriched between the DX-Se and DX groups, as shown in Figure 6. These pathways included primary bile acid biosynthesis, glycerophospholipid metabolism, biosynthesis of nucleotide sugars, arachidonic acid metabolism, amino sugar and nucleotide sugar metabolism, choline metabolism in cancer, tryptophan metabolism, and secondary bile acid biosynthesis. Among them, secondary bile acid biosynthesis is a significant enrichment pathway. Bile acids, hormone-like molecules synthesized by hepatocytes, activate nuclear receptors that regulate a wide array of metabolic activities [54], including glucose and fat metabolism [55], inflammation inhibition [56], and detoxification [57]. Secondary bile acids are generated by the metabolism of primary bile acids by microorganisms in the intestine, and have stronger lipophilicity and biological activity compared to primary bile acids. The physiological functions of secondary bile acids in the body include reducing cholesterol levels in the blood, regulating blood glucose levels, affecting cholesterol metabolism, and immune signaling. Secondary bile acids regulate lipid and glucose metabolism by activating receptors in the liver and intestine, such as TGR5 [58]. Related studies have shown that selenium (Se) is a micronutrient involved in important health functions and it has been suggested to shape gut microbiota [59]. Selenium-enriched peptides could ameliorate overweight gain, excess fat accumulation, serum lipid metabolism, and insulin resistance. The potential mechanism might be associated with the increase in thermogenesis, reduced oxidative stress, and inflammation, which regulated the gene expression in lipid and cholesterol metabolism. In addition, selenium-enriched peptides also maintained the intestinal integrity and modulated the gut microbiota [60]. Gema et al. found that Se may modulate the gut microbiota, specifically enhancing the bacterial activity that converts primary bile acids into secondary bile acids, thereby activating this pathway [61]. These secondary bile acids, in turn, function as hormone-like signaling molecules. They regulate lipid metabolism by activating nuclear receptors in the liver and intestine, which suppresses the activity of key enzymes for cholesterol synthesis and promotes cholesterol excretion into bile [61]. In this study, the addition of SEY to the diet modulated the secondary bile acid metabolism pathway in the chickens, resulting in reduced cholesterol levels. As shown in Figure S2A, omega–muricholic acid and allocholic acid are differential metabolites in the secondary bile acid biosynthesis. There are an extremely significant negative correlation (*p* < 0.01) and the most significant negative correlation (*p* < 0.001) between omega–muricholic acid and allocholic acid and cholesterol, respectively. Furthermore, by modulating the metabolism of secondary bile acids, the efficiency of fat digestion and absorption in the gut can be enhanced, resulting in increased release of free fatty acids [62,63]. These fatty acids act as key precursors that are further converted into VOCs during subsequent processing [64]. In the present study, the most significant positive correlation is between omega–muricholic acid and allocholic acid and VOCs such as 12-methyltridecanal, trans-2-heptenal, (Z)-3-hexenyl hexanoat, naphthalene, nonyl acetate, maltol, and alpha-terpinene, and an extremely significant negative correlation is found with VOCs such as 1-dodecanol, linalyl butyrate, and 4,5-Dimethylthiazole (Figure S2A). Among these, 12-methyltridecanal is described as having the odor of cooked meat, tallow, fat, meat broth, and sweat. It is generated when branched-chain fatty acid precursors deposited in chicken meat undergo oxidation during heating, and their resulting hydroperoxides undergo β-scission [65]. This VOC was reported to be a character impact component of goat meat analyzed by SPME [66] and may contribute to the “meaty” aroma of chicken meat. Therefore, in Dongxiang green-shell chickens, the secondary bile acid metabolism driven by SEY not only reduces cholesterol, but its related metabolic changes may also directly or indirectly promote the formation of these key meat flavor aroma substances, thereby synergistically improving the overall flavor quality of chicken meat.

**Figure 7:**
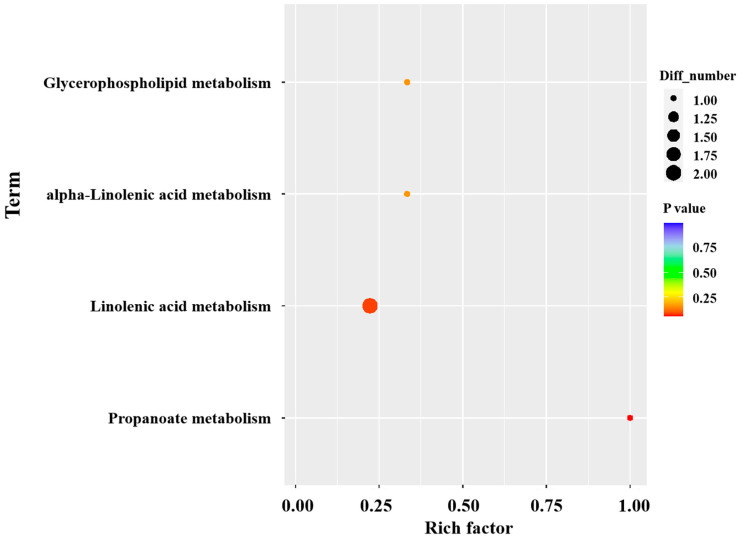
KEGG pathway analysis of chicken meat from Jingfen No. 1 laying hen.

KEGG pathway analysis of the differential metabolites revealed eight metabolic pathways enriched between the JF-Se and JF groups, as shown in Figure 7. These pathways included propanoate metabolism, linoleic acid metabolism, alpha-Linolenic acid metabolism, and glycerophospholipid metabolism. Among them, propanoate metabolism is a significant enrichment pathway. Propanoate is a type of short chain fatty acid that is mainly produced through fermentation by gut microbiota or tissue metabolism. Its metabolic pathway mainly includes the conversion of propanoate to acetyl CoA, entering the tricarboxylic acid cycle (TCA cycle), or participating in branched-chain amino acid metabolism. Gut microbiota may ferment carbohydrates (such as cellulose) in feed to produce propanoate, indirectly affecting muscle metabolism [67]. Cis-2-Methylaconitate is differential metabolite in the propanoate metabolism. As shown in Figure S2B, there is a significant negative correlation (*p* < 0.05) between cis-2-Methylaconitate and VOCs such as neral and cis-3-hexen-1-ol. Neral is described as having the odor of lemon, and cis-3-hexen-1-ol is described as having the odor of grass. This may be due to the combination of propionic acid and alcohols forming propanoate (such as ethyl propionate), which impart fruity or sweet aromas. Alternatively, propionic acid can be oxidized or decarboxylated to form acetaldehyde, which is further converted into other aldehydes such as hexanal, contributing to the odor of green grass or fat. Adding selenium-enriched yeast to the diet of Jingfen No. 1 laying hens may affect the muscle metabolism environment through propanoate metabolism, or indirectly regulate the production of sulfides (such as hydrogen sulfide) or nitrogen-containing compounds, increasing the grassy notes of chicken meat and reducing its gamey taste.

## 4. Conclusions

This study employed a multi-omics approach, integrating flavoromics and metabolomics, to investigate the impact of selenium-enriched yeast supplementation in hen diets on chicken meat flavor. GC-MS analysis detected ninety-eight VOCs, primarily aldehydes, alcohols, ketones, and esters. The calculation of OAVs identified key flavor contributors, including butyl acetate, hexanal, 1-pentanol, octanal, 1-hexanol, nonanal, 1-octen-3-ol, 1-heptanol, 1-octanol, 1-nonanol, and geranial. Compared to the control group, chicken meat from the SEY-treated group exhibited a more abundant and complex flavor profile, characterized by enhanced fatty, mushroom, fruity, and vanilla notes. Furthermore, SEY supplementation significantly improved the nutritional quality of the meat. It reduced the content of saturated fatty acids while increasing unsaturated fatty acids, and markedly lowered cholesterol levels in Dongxiang green-shell chicken. The treatment also positively influenced the amino acid composition, as evidenced by increased ratios of essential amino acids to total amino acids (EAA/TAA) and essential to non-essential amino acids (EAA/NEAA), indicating superior protein quality.

Metabolomics analysis elucidated potential mechanisms. In Dongxiang green-shell chickens, SEY appears to reduce cholesterol levels via the secondary bile acid biosynthesis pathway, and its related metabolic changes may also directly or indirectly promote the formation of these key meat flavor–aroma substances. In Jingfen No. 1 laying hens, SEY altered the muscle metabolism environment through propanoate metabolism, potentially indirectly regulating the production of sulfides (e.g., hydrogen sulfide) and nitrogen-containing compounds. This mechanism likely contributes to increased grassy notes and a reduction in the gamey taste.

These findings demonstrate the considerable potential of selenium-enriched yeast as a functional feed resource and provide valuable insights for its practical application in producing high-quality, flavorful chicken.

## Figures and Tables

**Table 1:** The identified VOCs and analysis of content in chicken meat coming from different groups (*n* = 6, data are presented as mean ± standard deviation).

No.	Compound	Odor Description	Content/ng/g				*p*-Value
			DX-Se	DX	JF-Se	JF	
1	Butyl acetate	pear	1810.03 ± 531.99	2136.44 ± 937.68	1487.32 ± 331.50	1595.55 ± 265.59	0.051
2	Hexanal	grass, tallow, fat	1221.01 ± 268.77 b	1092.00 ± 193.35 b	nd *	3446.35 ± 476.03 a	<0.001
3	2-Methylbutyl acetate	fruit	nd	291.15 ± 67.96	nd	nd	-
4	p-Xylene	sweet	121.60 ± 31.77 ab	140.59 ± 38.15 a	96.84 ± 16.15 b	104.98 ± 17.76 b	0.002
5	m-Xylene	plastic	164.51 ± 52.09 a	158.83 ± 25.20 a	95.27 ± 14.90 b	107.50 ± 21.23 b	<0.001
6	1-Butanol	medicine, fruit	726.40 ± 236.39 a	500.22 ± 180.03 b	374.10 ± 98.98 bc	298.62 ± 59.62 c	<0.001
7	1-Penten-3-ol	butter, pungent	417.58 ± 181.11 b	742.04 ± 247.98 a	358.02 ± 90.54 b	377.63 ± 104.20 b	<0.001
8	alpha-Terpinene	lemon	331.53 ± 65.18	nd	nd	nd	-
9	o-Xylene	geranium	147.75 ± 48.31 a	138.90 ± 17.90 a	68.36 ± 17.12 b	99.46 ± 19.11 b	<0.001
10	2-Heptanone	soap	nd	nd	nd	434.17 ± 36.65	-
11	3-Methyl-3-butenyl acetate	-	1658.68 ± 287.87 a	1430.58 ± 429.59 a	1621.29 ± 404.37 a	783.77 ± 66.62 b	<0.001
12	Limonene	lemon, orange	1353.80 ± 250.82 a	1028.29 ± 256.75 b	970.88 ± 253.78 b	582.81 ± 86.24 c	<0.001
13	2-Methyl-1-butanol	wine, onion, malt	1486.69 ± 368.86 a	1533.29 ± 348.90 a	744.96 ± 147.15 b	575.96 ± 85.71 b	<0.001
14	Isoamyl alcohol	whiskey, malt, burnt	892.65 ± 459.75 b	1696.21 ± 582.47 a	499.71 ± 107.56 c	491.42 ± 56.16 c	<0.001
15	2-Methylpyridine	sweat	560.12 ± 83.82	628.45 ± 213.46	nd	nd	0.313
16	Styrene	balsamic, gasoline	232.05 ± 56.24	214.01 ± 47.03	249.33 ± 49.77	273.77 ± 67.50	0.073
17	1-Pentanol	balsamic	1366.88 ± 329.89 b	2040.63 ± 695.08 a	722.07 ± 107.14 c	2192.49 ± 544.42 a	<0.001
18	3-Octanone	herb, butter, resin	671.70 ± 141.06 b	438.94 ± 57.51 c	495.04 ± 98.41 c	1608.21 ± 225.89 a	<0.001
19	Hexyl acetate	fruit, herb	nd	605.76 ± 133.16	nd	nd	-
20	4-Methylthiazole	roasted meat	nd	467.66 ± 84.66	nd	nd	-
21	2-Octanone	soap, gasoline	294.03 ± 58.32 b	nd	239.70 ± 23.61 b	844.42 ± 129.40 a	<0.001
22	Octanal	fat, soap, lemon, green	17,067.57 ± 3507.217 a	nd	nd	6529.79 ± 787.96 b	<0.001
23	trans-2-Penten-1-ol	mushroom	nd	nd	464.48 ± 57.39 b	1857.27 ± 655.44 a	<0.001
24	trans-2-Heptenal	soap, fat, almond	2566.85 ± 171.88	nd	nd	nd	-
25	1-Hexanol	resin, flower, green	2195.81 ± 593.00 ab	2862.71 ± 970.55 a	3429.51 ± 1055.22 a	3628.86 ± 1606.35 a	0.014
26	4,5-Dimethylthiazole	roast, smoke	158.65 ± 9.07 b	327.93 ± 26.61 a	nd	nd	<0.001
27	Heptyl acetate	pear, fruity, aromatic, sweet	nd	nd	nd	3194.82 ± 428.94	-
28	cis-3-Hexen-1-ol	grass	305.62 ± 64.93 c	502.56 ± 122.01 b	817.54 ± 334.69 a	nd	<0.001
29	2-Nonanone	hot milk, soap, green	nd	nd	10,467.27 ± 699.69	nd	-
30	2-Ethylhexyl acetate	fruit	728.53 ± 181.76 c	416.17 ± 55.88 d	1930.10 ± 282.89 b	2388.98 ± 137.31 a	<0.001
31	Nonanal	fat, citrus, green	764.73 ± 181.80 c	738.00 ± 172.49 c	1474.86 ± 580.12 b	4568.94 ± 746.25 a	<0.001
32	trans-3-Octen-2-one	nut, crushed bug	nd	nd	328.35 ± 62.61 b	1669.13 ± 116.17 a	<0.001
33	1,2,4,5-Tetramethylbenzene	rancid, sweet	22.02 ± 4.96 ab	25.94 ± 6.71 a	22.66 ± 2.71 ab	19.02 ± 4.51 b	0.015
34	trans-2-Octenal	green, nut, fat	nd	nd	252.97 ± 49.18 b	2206.44 ± 443.62 a	<0.001
35	(Z)-Linalool oxide	flower	nd	nd	1957.02 ± 857.52	nd	-
36	Acetic acid	sour	54,966.66 ± 5114.72	51,194.04 ± 7442.51	54,924.78 ± 9474.24	69,476.59 ± 18,431.33	0.392
37	1-Octen-3-ol	mushroom	4862.46 ± 395.34 b	nd	2952.10 ± 718.85 b	24,036.53 ± 1988.79 a	<0.001
38	1-Heptanol	chemical, green	1653.27 ± 477.39 c	4495.62 ± 618.97 ab	3834.73 ± 785.54 b	5096.65 ± 746.87 a	<0.001
39	Octyl acetate	coconut, vegetable oil, aromatic	896.32 ± 188.04 c	3779.71 ± 1143.82 a	4064.55 ± 423.03 a	2071.07 ± 328.49 b	<0.001
40	2-Ethylhexanol	rose, green	1001.08 ± 135.45 b	nd	nd	1559.82 ± 21.17 a	<0.001
41	Benzaldehyde	almond, burnt sugar	455.72 ± 91.87 b	638.17 ± 97.94 a	461.83 ± 86.99 b	387.99 ± 79.60 b	<0.001
42	(E)-2-Nonenal	cucumber, fat, green	nd	nd	21,343.19 ± 1255.49	nd	-
43	1-Octanol	chemical, metal, burnt	2328.84 ± 945.05 c	5081.59 ± 831.94 b	5815.75 ± 1007.00 b	8053.98 ± 1307.92 a	<0.001
44	Nonyl acetate	sweet, fruit	1349.61 ± 145.91	nd	nd	nd	-
45	Terpinen-4-ol	turpentine, nutmeg, must	nd	nd	9789.53 ± 1989.03	nd	-
46	2-Undecanone	orange, fresh, green	nd	nd	15,376.01 ± 2169.577 b	31,326.94 ± 4791.55 a	0.039
47	Phenylacetaldehyde	hawthorn, honey, sweet	nd	198.17 ± 3.99	nd	nd	-
48	(Z)-2-Decenal	tallow	7277.38 ± 819.50 a	6566.06 ± 285.38 b	nd	nd	0.010
49	1-Nonanol	fat, green	7750.55 ± 1210.23 bc	12,293.87 ± 1826.74 a	7515.57 ± 1013.92 c	9343.18 ± 1700.22 b	<0.001
50	(Z)-3-Hexenyl hexanoate	fruit, prune	11,557.08 ± 65.46 b	10,511.71 ± 1026.11 b	16,287.84 ± 2349.83 a	nd	<0.001
51	Isoamyl octanoate	fruity, orange, pear, melon	4657.59 ± 770.07	4804.05 ± 1049.69	4285.41 ± 1689.11	5112.82 ± 830.36	0.375
52	Decyl acetate	orange, oil	7370.33 ± 2229.59 c	9875.28 ± 1892.89 b	12,007.74 ± 554.22 a	nd	<0.001
53	Neral	lemon	9459.33 ± 1310.74 a	7782.53 ± 2466.56 b	6933.11 ± 1442.44 b	nd	0.007
54	Linalyl butyrate	pear, sweet	1455.47 ± 563.34 b	4044.05 ± 859.18 a	nd	nd	<0.001
55	(E,E)-2,4-nonadienal	fat, wax, green	nd	nd	2912.11 ± 945.50	nd	-
56	gamma-Caprolactone	coumarin, sweet	8912.09 ± 1932.89 b	nd	20,724.54 ± 6754.67 a	23,929.92 ± 6131.51 a	<0.001
57	3-Methyl-2,4-nonanedione	straw, fruit	5277.06 ± 174.49 c	7107.94 ± 644.46 c	40,609.36 ± 4491.47 a	23,809.75 ± 7967.48 b	<0.001
58	Neodihydrocarveol	-	nd	4031.13 ± 623.33	nd	nd	-
59	Neryl acetate	fruit	nd	7017.12 ± 1107.90	nd	nd	-
60	Geranial	lemon, mint	4214.65 ± 643.91 b	6370.90 ± 1584.24 a	4342.29 ± 204.12 b	nd	<0.001
61	Naphthalene	tar	47.75 ± 5.85	nd	nd	nd	-
62	trans-2-Undecenal	soap, fat, green	2604.32 ± 230.76 c	17,725.50 ± 2194.52 a	9059.91 ± 2283.96 b	9994.97 ± 944.88 b	<0.001
63	Methyl 2-nonynoate	green, floral, violet	nd	nd	5886.43 ± 970.74	6028.42 ± 493.02	0.659
64	1-Decanol	fat	4595.91 ± 296.38 b	nd	22,501.71 ± 8511.43 a	27,981.03 ± 7502.72 a	<0.001
65	Citronellol	rose	5806.27 ± 894.07 c	10,203.10 ± 3212.73 a	7767.29 ± 2214.03 b	8400.31 ± 1294.11 b	<0.001
66	gamma-Heptalactone	nut, fat, fruit	6497.89 ± 2079.67 c	12,191.69 ± 5505.41 b	19,015.56 ± 5830.05 a	15,490.80 ± 3568.64 ab	<0.001
67	Geranyl isobutyrate	floral	nd	126.18 ± 19.36	nd	nd	-
68	cis-Geranylacetone	-	nd	nd	138.64 ± 25.82 a	96.15 ± 14.28 b	<0.001
69	Capronic acid	sweat	32.12 ± 9.69 b	41.15 ± 11.41 b	44.37 ± 12.10 b	69.40 ± 17.83 a	<0.001
70	trans-p-Methane-8-thiol-3-one	-	nd	nd	nd	62.02 ± 7.99	-
71	alpha-Ionone	wood, violet	nd	5.86 ± 0.61	nd	nd	-
72	Neryl butyrate	-	nd	205.35 ± 66.16	nd	nd	-
73	1-Undecanol	mandarin	116.97 ± 40.46 b	181.21 ± 27.70 a	169.20 ± 5.39 a	nd	<0.001
74	cis-p-Methane-8-thiol-3-one	-	20.03 ± 0.67 b	nd	42.97 ± 5.42 a	54.05 ± 4.01 a	<0.001
75	12-Methyltridecanal	cooked meat, tallow, fat, meat broth, sweat	40.44 ± 4.52	nd	nd	nd	-
76	(E)-Whiskey lactone	flower, lactone	17.34 ± 8.42 c	51.82 ± 18.26 b	57.20 ± 14.29 b	70.38 ± 8.32 a	<0.001
77	gamma-Octalactone	coconut	nd	nd	1480.07 ± 153.22 a	998.90 ± 439.13 b	0.002
78	Butylated hydroxytoluene	musty	7.13 ± 0.96	nd	nd	nd	-
79	Tetradecanal	flower, wax	58.20 ± 14.98	nd	nd	nd	-
80	beta-Ionone	seaweed, violet, flower, raspberry	nd	nd	73.80 ± 10.41 b	84.57 ± 13.67 a	0.047
81	Maltol	caramel	464.57 ± 125.92	nd	nd	nd	-
82	Benzothiazole	gasoline, rubber	10.73 ± 2.10 b	13.91 ± 1.93 a	14.19 ± 2.92 a	14.26 ± 4.07 a	0.011
83	(E)-2-Hexenoic acid	must, fat	377.65 ± 50.00	nd	nd	nd	-
84	1-Dodecanol	fat, wax	25.46 ± 3.32 b	97.55 ± 32.45 a	nd	nd	<0.001
85	Caryophyllene oxide	herbal, sweet, spice	nd	nd	nd	1204.91 ± 117.96	-
86	(Z)-Nerolidol	wax	91.41 ± 42.53	124.94 ± 39.11	98.31 ± 34.53	103.19 ± 37.60	0.186
87	(E)-2-dodecen-1-ol	oil	nd	nd	112.46 ± 28.36 b	157.11 ± 35.07 a	0.003
88	4-Methoxybenzaldehyde	mint, sweet	7.03 ± 0.99 b	nd	22.67 ± 5.98 a	22.71 ± 4.27 a	<0.001
89	gamma-Nonalactone	coconut, peach	5.21 ± 1.56 c	44.24 ± 16.03 b	118.76 ± 37.66 a	140.20 ± 22.12 a	<0.001
90	Pentadecanal	fresh	nd	nd	72.28 ± 6.84 b	87.62 ± 12.03 a	0.001
91	Caprylic acid	sweat, cheese	nd	7.96 ± 2.00	8.16 ± 0.89	nd	0.768
92	Hexadecanal	cardboard	17.20 ± 2.94 b	19.94 ± 4.97 b	39.50 ± 9.46 a	45.37 ± 16.32 a	<0.001
93	gamma-Decalactone	peach, fat	nd	nd	53.33 ± 8.33	61.40 ± 11.63	0.072
94	Nonanoic acid	green, fat	31.13 ± 1.73 a	15.01 ± 2.84 b	14.46 ± 1.70 b	11.08 ± 0.88 c	<0.001
95	1-Tetradecanol	coconut	14.83 ± 2.50	nd	nd	nd	-
96	Massoia lactone	peach	nd	nd	9.15 ± 4.05	11.16 ± 4.32	0.264
97	Capric acid	fat	nd	13.54 ± 3.14 b	11.48 ± 4.21 b	19.30 ± 3.56 a	<0.001
98	1-Hexadecanol	wax, flower	8.70 ± 1.53 a	nd	5.21 ± 1.12 b	nd	<0.001

* nd means no detected; odor description: cited from the website: http://flavornet.org/flavornet.html (accessed on 8 November 2025); and “-” means no value. Data with different letters in a row are significantly different (*p* < 0.05).

**Table 2:** OAVs of VOCs detected in different chickens.

NO.	Compound	OT * (ng/g)	OAV			
			DX-Se	DX	JF-Se	JF
1	Butyl acetate	58	31.21	36.84	25.64	27.51
2	Hexanal	5	244.20	218.40	nd	689.27
4	p-Xylene	490	0.25	0.29	0.20	0.21
6	1-Butanol	459.2	1.58	1.09	0.81	0.65
14	Isoamyl alcohol	8100	0.11	0.21	0.06	0.06
17	1-Pentanol	1	1366.88	2040.63	722.07	2192.49
22	Octanal	0.7	24,382.24	nd	nd	9328.27
25	1-Hexanol	1	2195.81	2862.71	3429.51	3628.86
28	cis-3-Hexen-1-ol	200	1.53	2.51	4.09	nd
29	2-Nonanone	38.9	nd	nd	269.08	nd
31	Nonanal	1.1	695.21	670.91	1340.78	4153.58
37	1-Octen-3-ol	1	4862.46	nd	2952.10	24,036.53
38	1-Heptanol	1	1653.27	4495.62	3834.73	5096.65
41	Benzaldehyde	350	1.30	1.82	1.32	1.11
43	1-Octanol	1	2328.84	5081.59	5815.75	8053.98
47	Phenylacetaldehyde	4	nd	49.54	nd	nd
49	1-Nonanol	45.5	170.34	270.19	165.18	205.34
60	Geranial	32	131.71	199.09	135.70	nd
80	beta-Ionone	6	nd	nd	12.30	14.10
82	Benzothiazole	8	1.34	1.74	1.77	1.78
84	1-Dodecanol	82	0.31	1.19	nd	nd

* OT is the odor threshold of the VOCs in water, which was obtained from book [29]; nd means no detected.

**Table 3:** Compositions of fatty acid (%) present in the chicken meat (*n* = 6).

Fatty Acids	DX-Se	DX	JF-Se	JF	*p*-Value
C4:0	0.31 ± 0.04 b	0.27 ± 0.06 b	0.34 ± 0.19 b	1.18 ± 0.62 a	0.048
C6:0	1.30 ± 0.76 a	1.41 ± 1.23 a	1.37 ± 0.83 a	1.78 ± 0.88 a	0.650
C8:0	0.76 ± 0.43 a	0.98 ± 0.87 a	0.93 ± 0.54 a	1.28 ± 0.48 a	0.485
C10:0	0.54 ± 0.47 a	0.87 ± 0.80 a	0.41 ± 0.25 a	1.10 ± 0.54 a	0.287
C12:0	0.61 ± 0.19 a	1.38 ± 0.35 a	0.72 ± 0.18 a	1.13 ± 0.60 a	0.138
C13:0	0.62 ± 0.23 a	0.81 ± 0.50 a	0.59 ± 0.25 a	1.31 ± 0.86 a	0.072
C14:0	0.56 ± 0.12 a	0.59 ± 0.16 a	0.85 ± 0.59 a	0.51 ± 0.16 a	0.060
C15:0	1.31 ± 0.56 a	1.96 ± 0.65 a	1.34 ± 0.58 a	2.05 ± 1.20 a	0.387
C16:0	22.41 ± 2.26 a	22.89 ± 2.55 a	24.42 ± 1.21 a	24.26 ± 3.34 a	0.331
C17:0	0.32 ± 0.20 a	0.33 ± 0.21 a	0.35 ± 0.26 a	0.44 ± 0.25 a	0.703
C18:0	8.60 ± 1.30 a	9.22 ± 1.56 a	9.03 ± 3.63 a	9.60 ± 1.03 a	0.793
C20:0	0.15 ± 0.08 a	0.17 ± 0.11 a	0.16 ± 0.10 a	0.29 ± 0.22 a	0.116
C21:0	0.20 ± 0.07 a	0.16 ± 0.05 a	0.16 ± 0.10 a	0.29 ± 0.22 a	0.129
Σ SFA	37.69	41.04	40.67	45.22	
C14:1	1.04 ± 0.03 b	1.76 ± 0.88 ab	1.04 ± 0.55 b	2.27 ± 1.09 a	0.011
C15:1	0.14 ± 0.03 a	0.28 ± 0.12 a	0.21 ± 0.15 a	0.39 ± 0.11 a	0.203
C16:1	3.56 ± 0.79 a	3.13 ± 0.77 a	3.14 ± 0.55 a	3.00 ± 0.73 a	0.290
C17:1	0.30 ± 0.07 ab	0.16 ± 0.08 b	0.13 ± 0.08 b	0.44 ± 0.31 a	0.043
C18:1	39.58 ± 4.44 a	37.55 ± 3.99 a	39.25 ± 3.06 a	33.70 ± 5.59 a	0.369
C20:1	0.28 ± 0.11 a	0.26 ± 0.06 a	0.28 ± 0.09 a	0.33 ± 0.06 a	0.276
C22:1	0.15 ± 0.02 a	0.14 ± 0.03 a	0.14 ± 0.02 a	0.11 ± 0.04 a	0.235
C24:1	0.13 ± 0.03 b	0.16 ± 0.04 b	0.14 ± 0.05 b	0.29 ± 0.17 a	0.049
Σ MUFA	45.18	42.90	44.33	40.53	
C18:2	15.53 ± 3.17 a	14.42 ± 1.56 a	13.28 ± 4.14 a	12.16 ± 1.69 a	0.621
C20:2	0.12 ± 0.04 a	0.13 ± 0.03 a	0.17 ± 0.04 a	0.13 ± 0.05 a	0.146
C22:2	0.20 ± 0.10 b	0.20 ± 0.07 b	0.18 ± 0.11 b	0.35 ± 0.14 a	0.028
C18:3	0.49 ± 0.15 a	0.53 ± 0.13 a	0.51 ± 0.25 a	0.57 ± 0.16 a	0.747
C20:4	0.56 ± 0.37 b	0.63 ± 0.32 b	0.67 ± 0.35 b	0.81 ± 0.43 b	0.501
C22:6	0.23 ± 0.11 b	0.21 ± 0.18 b	0.19 ± 0.14 b	0.45 ± 0.29 a	0.035
Σ PUFA	17.13	16.05	14.99	14.25	

Data with different letters in a row are significantly different (*p* < 0.05).

**Table 4:** Amino acid analysis of the chicken meat (*n* = 6).

Amino Acids (g/100 g)		DX-Se	DX	JF-Se	JF	*p*-Value
Bitter amino acids	His	0.85 ± 0.08 a	0.88 ± 0.20 a	0.83 ± 0.08 a	0.75 ± 0.10 a	0.126
	Arg	1.42 ± 0.11 a	1.40 ± 0.08 a	1.49 ± 0.13 a	1.39 ± 0.13 a	0.218
	Tyr	0.78 ± 0.06 a	0.78 ± 0.05 a	0.82 ± 0.07 a	0.77 ± 0.07 a	0.175
	Val	1.19 ± 0.08 a	1.15 ± 0.09 a	1.22 ± 0.11 a	1.13 ± 0.10 a	0.242
	Lys	1.94 ± 0.15 a	1.93 ± 0.13 a	2.02 ± 0.16 a	1.90 ± 0.15 a	0.271
	Ile	1.13 ± 0.09 a	1.09 ± 0.07 a	1.17 ± 0.11 a	1.09 ± 0.10 a	0.188
	Leu	1.78 ± 0.14 a	1.75 ± 0.11 a	1.85 ± 0.16 a	1.73 ± 0.15 a	0.260
	Phe	1.12 ± 0.07 ab	1.10 ± 0.08 ab	1.16 ± 0.10 a	1.05 ± 0.09 b	0.049
Sweet amino acids	Ser	0.76 ± 0.06 ab	0.78 ± 0.06 ab	0.79 ± 0.07 a	0.72 ± 0.06 b	0.044
	Gly	0.96 ± 0.06 a	0.95 ± 0.07 a	1.00 ± 0.08 a	0.94 ± 0.09 a	0.302
	Thr	0.94 ± 0.07 a	0.95 ± 0.06 a	0.98 ± 0.08 a	0.90 ± 0.07 a	0.162
	Ala	1.34 ± 0.10 a	1.32 ± 0.10 a	1.40 ± 0.13 a	1.30 ± 0.12 a	0.221
	Pro	0.80 ± 0.09 a	0.76 ± 0.06 a	0.79 ± 0.08 a	0.75 ± 0.08 a	0.437
Umami amino acids	Glu	3.23 ± 0.28 a	3.21 ± 0.21 a	3.39 ± 0.29 a	3.20 ± 0.28 a	0.349
	Asp	2.01 ± 0.15 a	1.99 ± 0.15 a	2.09 ± 0.17 a	1.95 ± 0.16 a	0.267
Sulfur-containing amino acids	Met	0.69 ± 0.08 a	0.66 ± 0.04 a	0.70 ± 0.08 a	0.65 ± 0.07 a	0.325
EAA (g/100 g)		8.79	8.63	9.1	8.44	
TAA (g/100 g)		20.94	20.7	21.7	20.21	
EAA/TAA/%		41.98	41.69	41.94	41.76	
EAA/NEAA/%		72.35	71.50	72.22	71.71	

Data with different letters in a row are significantly different (*p* < 0.05).

## Data Availability

The original contributions presented in the study are included in the article/Supplementary Material, further inquiries can be directed to the corresponding author.

## Supplementary Materials

The following supporting information can be downloaded at: https://www.mdpi.com/article/10.3390/foods14234060/s1, Table S1: Composition and nutrient levels of the basal diet (as-fed basis); Table S2: The identified VOCs of chicken; Figure S1: (A) Principal components analysis (PCA) score plot of VOCs in chicken coming from different groups, (B) Loading plot of VOCs in chicken coming from different groups; Figure S2: (A) Spearman correlation of differential metabolites with characteristic VOCs and partial nutritional quality of chicken meat from Dongxiang green-shell chicken, (B) Spearman correlation of differential metabolites with characteristic VOCs and partial nutritional quality of chicken meat from Jingfen No. 1 laying hen; Excel: Metabolite Information.
