# Supplementary material for: Metabolic Pathway Analysis in Chicken Induced by Selenium-Enriched Yeast: Insights from Flavoromics and Metabolomics
Source: Foods. 2025 Nov 26;14(23):4060. doi: 10.3390/foods14234060 (PMC12692524; doi:10.3390/foods14234060)
Supplement: Supplementary file 1 [file foods-14-04060-s001.zip › Supplementary file.pdf]

Metabolic Pathway Analysis in Chicken Induced by Selenium-Enriched Yeast: Insights from Flavoromics and Metabolomics

Dan Fei [1,2], Min Xie [1,2], Daojie Li [1,2], Yelan Guang [1,2] and Yaomin Zhou [1,2,*]

1 Institute for Quality, Safety and Standard of Agricultural Products, Jiangxi Academy of Agricultural Sciences, Nanchang 330200, China

2 Jiangxi Provincial Key Laboratory for Quality and Safety of Agricultural Products, Nanchang 330200, China

* Correspondence: zhouyaomin666@163.com; Tel.: +86-13970932355

Table S1. Composition and nutrient levels of the basal diet (as fed basis).

Ingredients	Composition (%)	Nutrient	Nutrient levels
Corn	60	Crude protein (%)	16.5
Soybean meal	25	Calcium (%)	3.26
Wheat bran	5	Total phosphorus (%)	0.44
Limestone	5	Available phosphorus (%)	0.21
Premix	5	Sodium chloride (%)	0.33
		Lysine (%)	0.911
		Methionine (%)	0.369
		Methionine + cystine (%)	0.585
		Threonine (%)	0.676
		Tryptophan (%)	0.206
		Metabolizable energy (Kcal/kg)	2611
		Crude fiber (%)	2.7
		Crude fat (%)	2.8
		Crude ash (%)	12.1

TableS2. The identified VOCs of chicken.

No.	Retention time / min	Compound	Formula	Retention index (theoretical value)*	Retention index (measurement value)	Matching percentage /%
**1**	4.038	Butyl acetate	$C_6H_{12}O_2$	1059	1052	86.5
**2**	4.118	Hexanal	$C_6H_{12}O$	1063	1062	90.2
**3**	4.316	2-Methylbutyl acetate	$C_7H_{14}O_2$	1073	1071	83.7
**4**	5.481	p-Xylene	C_8H_{10}	1124	1124	88.6
**5**	5.638	m-Xylene	C_8H_{10}	1130	1127	85.2
**6**	5.795	1-Butanol	$C_4H_{10}O$	1136	1145	87.4
**7**	6.109	1-Penten-3-ol	$C_5H_{10}O$	1148	1150	82.3
**8**	6.633	alpha-Terpinene	$C_{10}H_{16}$	1168	1169	84.2
**9**	6.711	o-Xylene	C_8H_{10}	1171	1171	80.7
**10**	6.737	2-Heptanone	$C_7H_{14}O$	1172	1174	84.6
**11**	7.052	3-Methyl-3-butenyl acetate	$C_7H_{12}O_2$	1184	1185	88.1
**12**	7.130	Limonene	$C_{10}H_{16}$	1187	1187	89.5
**13**	7.392	2-Methyl-1-butanol	$C_5H_{12}O$	1197	1193	80.7
**14**	7.418	Isoamyl alcohol	$C_5H_{12}O$	1198	1198	94.3
**15**	7.419	2-Methylpyridine	C_6H_7N	1198	1198	87.2
**16**	8.294	Styrene	C_8H_8	1239	1245	86.4
**17**	8.379	1-Pentanol	$C_5H_{12}O$	1243	1246	82.7
**18**	8.484	3-Octanone	$C_8H_{16}O$	1248	1249	92.4
**19**	8.843	Hexyl acetate	$C_8H_{16}O_2$	1265	1266	88.5
**20**	8.885	4-Methylthiazole	C_4H_5NS	1267	1268	83.4
**21**	9.075	2-Octanone	$C_8H_{16}O$	1276	1276	92.7
**22**	9.160	Octanal	$C_8H_{16}O$	1280	1281	90.8
**23**	9.600	trans-2-Penten-1-ol	$C_5H_{10}O$	1301	1301	86.4
**24**	9.724	trans-2-Heptenal	$C_7H_{12}O$	1308	1307	87.2
**25**	10.433	1-Hexanol	$C_6H_{14}O$	1348	1346	91.8
**26**	10.468	4,5-Dimethylthiazole	$C_8H_{11}NO$	1350	1348	79.8
**27**	10.752	Heptyl acetate	$C_9H_{18}O_2$	1366	1368	88.6
**28**	10.893	cis-3-Hexen-1-ol	$C_6H_{12}O$	1374	1374	80.4
**29**	10.946	2-Nonanone	$C_9H_{18}O$	1377	1378	83.5
**30**	10.982	2-Ethylhexyl acetate	$C_{10}H_{20}O_2$	1379	1384	87.2
**31**	11.053	Nonanal	$C_9H_{18}O$	1383	1385	80.9
**32**	11.212	trans-3-Octen-2-one	$C_8H_{14}O$	1392	1396	81.5
**33**	11.494	1,2,4,5-Tetramethylbenzene	$C_{10}H_{14}$	1409	1410	83.4
**34**	11.572	trans-2-Octenal	$C_8H_{14}O$	1414	1417	78.8
**35**	11.820	(Z)-Linalool oxide	$C_{10}H_{18}O_2$	1430	1432	81.3
**36**	11.882	Acetic acid	$C_2H_4O_2$	1434	1434	89.1
**37**	12.038	1-Octen-3-ol	$C_8H_{16}O$	1444	1443	85.2
**38**	12.100	1-Heptanol	$C_7H_{16}O$	1448	1449	86.4
**39**	12.442	Octyl acetate	$C_{10}H_{20}O_2$	1470	1471	80.5

**40**	12.551	2-Ethylhexanol	$C_8H_{18}O$	1477	1482	76.9
**41**	12.979	Benzaldehyde	C_7H_6O	1505	1508	84.3
**42**	13.176	(E)-2-Nonenal	$C_9H_{16}O$	1519	1520	81.9
**43**	13.556	1-Octanol	$C_8H_{18}O$	1546	1546	86.7
**44**	13.923	Nonyl acetate	$C_{11}H_{22}O_2$	1572	1575	84.9
**45**	14.120	Terpinen-4-ol	$C_{10}H_{18}O$	1586	1590	77.5
**46**	14.148	2-Undecanone	$C_{11}H_{22}O$	1588	1592	79.4
**47**	14.721	Phenylacetaldehyde	C_8H_8O	1631	1632	81.3
**48**	14.761	(Z)-2-Decenal	$C_{10}H_{18}O$	1634	1634	76.8
**49**	14.839	1-Nonanol	$C_9H_{20}O$	1640	1639	83.5
**50**	14.956	(Z)-3-Hexenyl hexanoate	$C_{12}H_{22}O_2$	1649	1652	86.4
**51**	15.008	Isoamyl octanoate	$C_{13}H_{26}O_2$	1653	1655	88.7
**52**	15.256	Decyl acetate	$C_{12}H_{24}O_2$	1672	1667	79.4
**53**	15.256	Neral	$C_{10}H_{16}O$	1672	1679	78.1
**54**	15.334	Linalyl butyrate	$C_{14}H_{24}O_2$	1678	1680	81.2
**55**	15.489	(E,E)-2,4-nonadienal	$C_9H_{14}O$	1690	1689	80.6
**56**	15.490	gamma-Caprolactone	$C_6H_{10}O_2$	1690	1690	85.9
**57**	15.694	3-Methyl-2,4-nonanedione	$C_{10}H_{18}O_2$	1706	1700	82.4
**58**	15.743	Neodihydrocarveol	$C_{10}H_{18}O$	1710	1711	87.5
**59**	15.780	Neryl acetate	$C_{12}H_{20}O_2$	1713	1713	76.8
**60**	15.890	Geranial	$C_{10}H_{16}O$	1722	1724	90.1
**61**	15.939	Naphthalene	$C_{10}H_8$	1726	1730	89.7
**62**	16.135	trans-2-Undecenal	$C_{11}H_{20}O$	1742	1743	85.5
**63**	16.160	Methyl 2-nonynoate	$C_{10}H_{16}O_2$	1744	1745	90.3
**64**	16.245	1-Decanol	$C_{10}H_{22}O$	1751	1753	91.5
**65**	16.307	Citronellol	$C_{10}H_{20}O$	1756	1755	87.4
**66**	16.588	gamma-Heptalactone	$C_7H_{12}O_2$	1779	1788	80.6
**67**	16.845	Geranyl isobutyrate	$C_{14}H_{24}O_2$	1800	1802	83.4
**68**	17.020	cis-Geranylacetone	$C_{13}H_{22}O$	1815	1812	75.6
**69**	17.136	Capronic acid	$C_6H_{12}O_2$	1825	1828	89.2
**70**	17.217	trans-p-Methane-8-thiol-3-one	$C_{12}H_{20}O_2S$	1832	1836	86.4
**71**	17.345	alpha-Ionone	$C_{13}H_{20}O$	1843	1844	82.3
**72**	17.415	Neryl butyrate	$C_{14}H_{24}O_2$	1849	1850	88.9
**73**	17.461	1-Undecanol	$C_{11}H_{24}O$	1853	1851	84.6
**74**	17.554	cis-p-Methane-8-thiol-3-one	$C_{12}H_{20}O_2S$	1861	1863	85.3
**75**	17.601	12-Methyltridecanal	$C_{14}H_{28}O$	1865	1868	90.4
**76**	17.612	(E)-Whiskey lactone	$C_9H_{16}O_2$	1866	1870	77.4
**77**	17.984	gamma-Octalactone	$C_8H_{14}O_2$	1898	1900	77.0
**78**	18.018	Butylated hydroxytoluene	$C_{15}H_{24}O$	1901	1905	79.8
**79**	18.140	Tetradecanal	$C_{14}H_{28}O$	1912	1917	80.5
**80**	18.306	beta-Ionone	$C_{13}H_{20}O$	1927	1928	83.2
**81**	18.439	Maltol	$C_6H_6O_3$	1939	1932	84.9

**82**	18.461	Benzothiazole	C_7H_5NS	1941	1946	76.8
**83**	18.505	(E)-2-Hexenoic acid	$C_6H_{10}O_2$	1945	1944	82.5
**84**	18.561	1-Dodecanol	$C_{12}H_{26}O$	1950	1951	80.4
**85**	18.782	Caryophyllene oxide	$C_{15}H_{24}O$	1970	1972	91.2
**86**	18.948	(Z)-Nerolidol	$C_{15}H_{26}O$	1985	1983	88.6
**87**	19.177	(E)-2-dodecen-1-ol	$C_{12}H_{24}O$	2006	2000	84.2
**88**	19.199	4-Methoxybenzaldehyde	$C_8H_8O_2$	2008	2009	78.3
**89**	19.220	gamma-Nonalactone	$C_9H_{16}O_2$	2010	2012	82.4
**90**	19.305	Pentadecanal	$C_{15}H_{30}O$	2018	2023	77.6
**91**	19.474	Caprylic acid	$C_8H_{16}O_2$	2034	2036	80.4
**92**	20.437	Hexadecanal	$C_{16}H_{32}O$	2126	2130	85.4
**93**	20.457	gamma-Decalactone	$C_{10}H_{18}O_2$	2128	2127	83.1
**94**	20.579	Nonanoic acid	$C_9H_{18}O_2$	2140	2138	82.9
**95**	20.762	1-Tetradecanol	$C_{14}H_{30}O$	2158	2165	87.4
**96**	21.275	Massoia lactone	$C_{10}H_{16}O_2$	2209	2193	79.4
**97**	21.665	Capric acid	$C_{10}H_{20}O_2$	2249	2253	85.9
**98**	22.761	1-Hexadecanol	$C_{16}H_{34}O$	2364	2367	79.6

*Retention index (theoretical value) cited from the databases including the NIST 17, AROMA spectral database, and Shimadzu Off-flavor database.

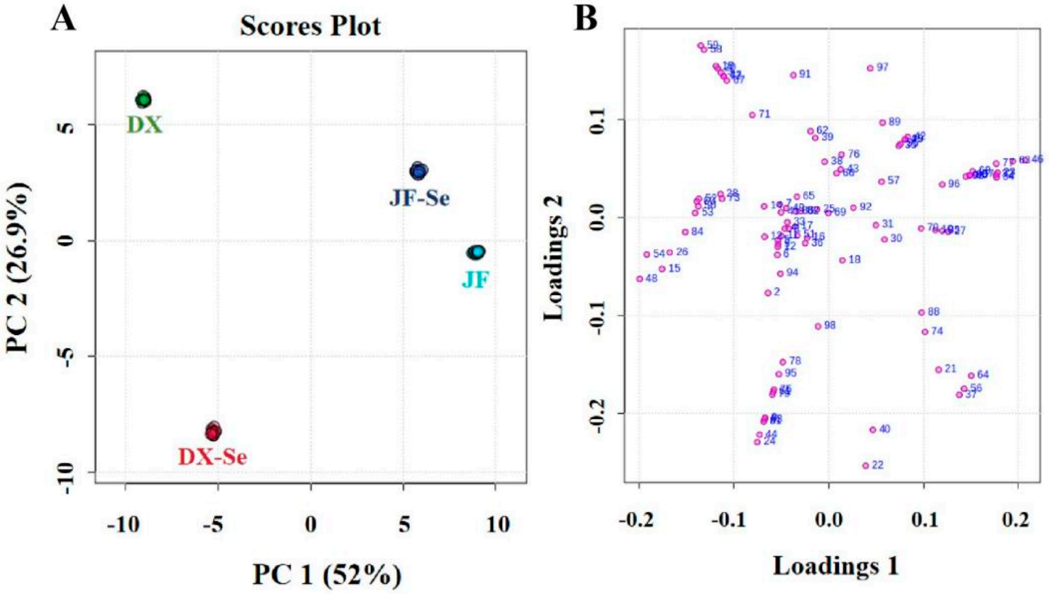

Figure S1. Principal components analysis (PCA) score plot of VOCs in chicken coming from different groups (A); Loading plot of VOCs in chicken coming from different groups (B). DX-Se: treatment group of Dongxiang green-shell chicken. DX: control group of Dongxiang green-shell chicken. JF-Se: treatment group of Jingfen laying hens. JF: control group of Jingfen laying hens.

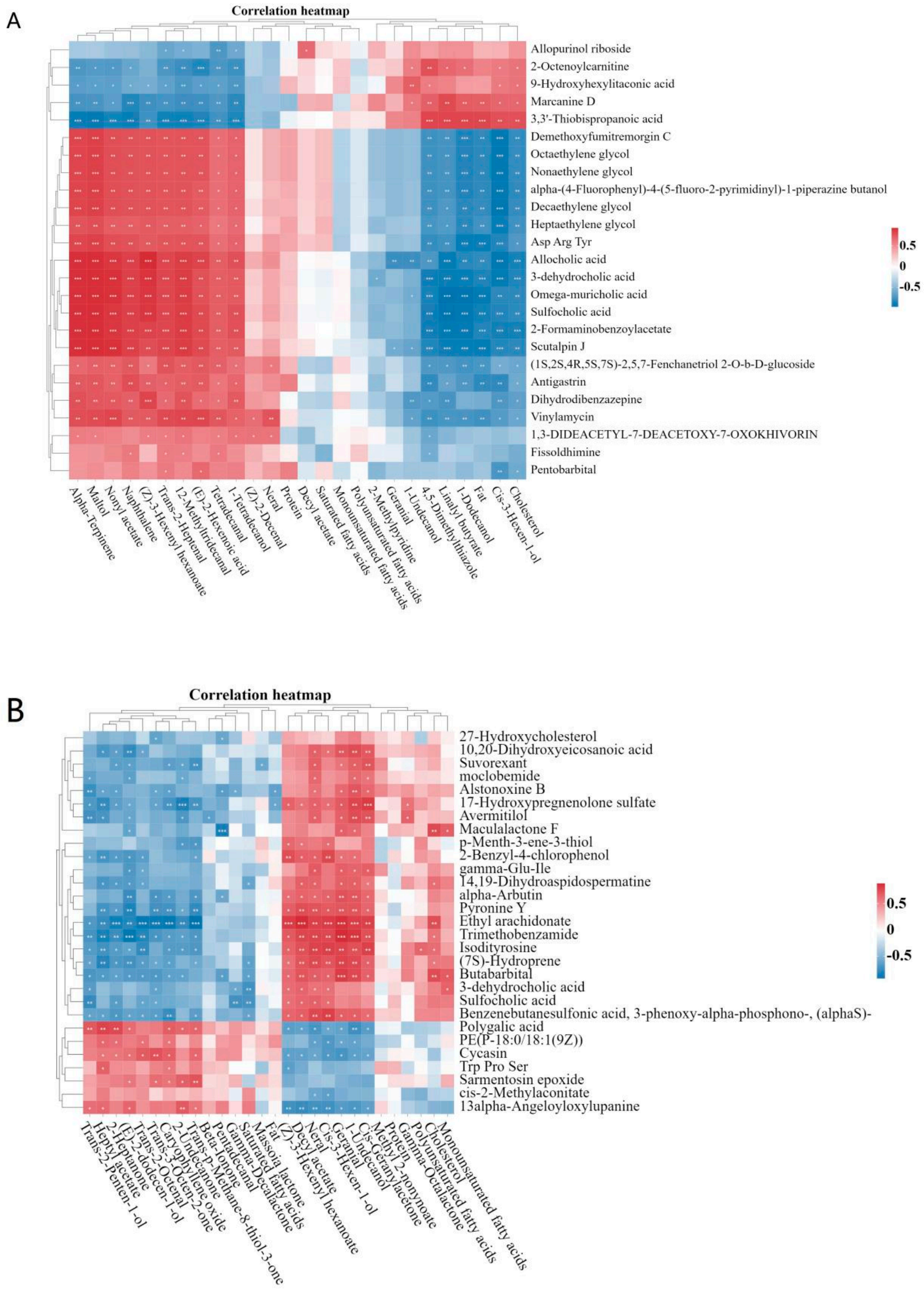

Figure S2. Spearman correlation of differential metabolites with characteristic VOCs and partial nutritional quality of chicken meat from Dongxiang green-shell chicken (A); Spearman correlation of differential metabolites with characteristic VOCs and partial nutritional quality of chicken meat from Jingfen No. 1 laying hen (B); * $P < 0.05$, ** $P < 0.01$, *** $P < 0.001$.
